# Identification of a Differentially Expressed TIR-NBS-LRR Gene in a Major QTL Associated to Leaf Rust Resistance in *Salix*

**DOI:** 10.1371/journal.pone.0168776

**Published:** 2016-12-21

**Authors:** Tom Martin, Ann-Christin Rönnberg-Wästljung, Jan Stenlid, Berit Samils

**Affiliations:** 1 Department of Plant Biology, Swedish University of Agricultural Sciences, Uppsala, Sweden; 2 Department of Forest Mycology and Plant Pathology, Swedish University of Agricultural Sciences, Uppsala, Sweden; New South Wales Department of Primary Industries, AUSTRALIA

## Abstract

An earlier identified major quantitative trait locus for resistance towards the willow leaf rust fungus *Melampsora larici-epitea* in a *Salix viminalis* x (*S*. *viminalis* × *S*. *schwerinii*) population was used to identify potential resistance genes to the rust pathogen. Screening a genomic bacterial artificial chromosome library with markers from the peak position of the QTL region revealed one gene with TIR-NBS-LRR (Toll Interleukin1 Receptor-Nucleotide Binding Site-Leucine-Rich Repeat) domain structure indicative of a resistance gene. The resistance gene analog was denoted RGA1 and further analysis revealed a number of non-synonymous single nucleotide polymorphisms in the LRR domain between the resistant and susceptible *Salix* genotypes. Gene expression levels under controlled conditions showed a significantly lower constitutive expression of RGA1 in the susceptible genotype. In addition, the susceptible genotype showed a significantly reduced expression level of the RGA1 gene at 24 hours post inoculation with *M*. *larici-epitea*. This indicates that the pathogen may actively suppress RGA1 gene expression allowing a compatible plant-pathogen interaction and causing infection.

## Introduction

The rapid growth, ease of clonal propagation from cuttings, phytoremediation properties, and ability to grow on land unsuitable for food crops make willow (*Salix* spp.) in short-rotation cultivation an excellent bioenergy crop. Willow is regarded as a carbon neutral source of biofuel and can be used in exchange for coal or other fossil fuels to generate energy for heat and electricity [1].

One of the major threats to a high and stable production of short-rotation willow is leaf rust, caused by the fungus *Melampsora larici-epitea* Kleb. The pathogen can damage leaf tissue, reduce the photosynthetic potential and cause premature leaf fall. In recent breeding programs a number of willow varieties with complete or almost complete resistance have been produced [2]. However, like many other rust fungi, *M*. *larici-epitea* has a high capability to overcome resistance [3–4] and cases of resistance breakdown in willow have already been experienced [5–6].

Willow (*Salix*) and poplar (*Populus*) are both members of the family Salicaceae and they share many ecological as well as genetic and genomic characteristics [7]. Although the genera separated around 45 million years ago (mya), the relatively slow rate of genomic change between willow and poplar implies that genomic resources in poplar are also useful in willow research [7]. In *P*. *trichocarpa* large scale analysis of resistance genes showed that they are numerous (over 400) and diverse [8–9].

The most represented group of resistance genes (R-genes) are those containing a nucleotide-binding-site (NBS) and leucine-rich-repeats (LRR), where the LRR domains have an important role for pathogen recognition specificity [10]. These genes often occur in clusters at specific loci and evolve through gene duplication, divergent selection and birth-and-death processes leading to highly adaptable structure domains with large differences between even closely related species [11]. The pathogen will, in turn, evolve new ways to avoid recognition, attack the plant and supress defence, which leads to the rapid evolution of both plant resistance genes and pathogen virulence genes in a biological arms race [12].

Our current pre-breeding research on rust resistance in short-rotation willow aims at introducing a diversity of resistance genes into marketed willow varieties with the purpose to achieve more long-lasting resistance. One of the most utilized genetic sources for rust resistance in short-rotation willow in Europe today is *S*. *schwerinii* Wolf, a species introduced from northern Asia with a high level of resistance to willow leaf rust in Europe. In order to select for diversity in resistance genes it is necessary to distinguish the resistance genes inherited from *S*. *schwerinii* from other resistance sources. When a major resistance gene is present it can mask the phenotypic expression of other resistance genes, which will go undetected in the selection process in conventional breeding. Identification of candidate resistance genes enables development of DNA markers that can be used for selection of resistant genotypes.

A major quantitative trait loci (QTL) which associated to field rust resistance and a number of resistance components (i.e. uredinia number, uredinia size, latent period, necrotic flecking), accounting for 15–56% of the resistant phenotype, was previously identified on linkage group Ib (LG Ib) in mapping population S_1_ from a cross *S*. *viminalis* L. × (*S*. *viminalis* × *S*. *schwerinii*) [13]. The aim of the present work was to analyse the region within this major QTL and to find the genes that underlie the resistant phenotype. We constructed a genomic BAC library of the hybrid *S*. *viminalis × S*. *schwerinii* to identify potential resistance genes. The available *P*. *trichocarpa* (Torr. & Gray) genomic resources, in combination with the recently released genome sequence of *S*. *pupurea* L., allowed a broader assessment of the region surrounding the QTL. We identified one gene that contained a typical resistance gene domain structure and was differentially expressed in the susceptible and resistant genotypes and down-regulated 24 hours post inoculation in a compatible interaction.

## Material and Methods

### Genomic region of interest (QTL)

**Fig 1:**
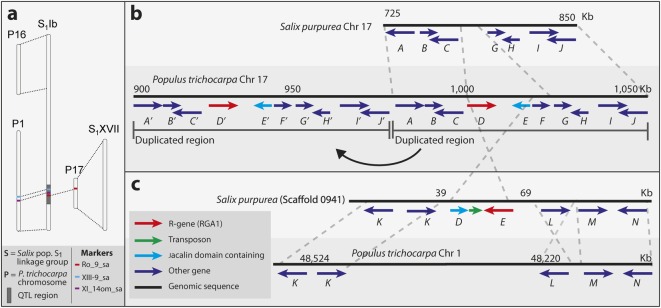
Graphical representation the genomic location of the QTL and RGA1 gene. (a) Location of the QTL on the *Salix* linkage group S_1_Ib. General similarities in chromosomal regions to *Populus trichocarpa* identified by Berlin et al. [7] are shown. Specific positions of markers XII-9_sa, XI_14om_sa and Ro_9_sa are highlighted. (b) Conservation between chromosome 17 regions in *Salix pupurea* and *P*. *trichocarpa*. Arrows represent hypothetical genes. A duplicated region containing ten genes (*A-J*) on poplar chromosome 17 is shown. Homologous genes in (b) and (c) are labelled with the same letter and/or colour. (c) *S*. *pupurea* Scaffold 0941 and *P*. *trichocarpa* chromosome 1 regions showing homologous genes (*K-N*). Function/products of genes *A-N*: *A* = Diacylglycerol acyltransferase, *B* = Transferase, *C* = Diacylglycerol acyltransferase, *D* = TIR-NBS-LRR resistance protein, *E* = Jacalin-like lectin domain, *F* = Ribosome inactivating protein, *G* = F-box domain, *H* = Plastocyanin-like domain, *I* = Transcription-coupled repair protein, *J* = Predicted transcriptional regulator, *K* = Transferase, *L* = IQ calmodulin-binding motif, *M* = Electron transporter, *N* = NAC transcription factor.

The major rust resistance QTL investigated in this study was previously identified in *Salix* mapping population S_1_, a hybrid cross between *S*. *viminalis* cv. ‘78183’ and *S*. *viminalis* × *S*. *schwerinii* cv. ‘Björn’ [13–14]. ‘Björn’ is highly resistant to rust infections whereas ‘78183’ is susceptible [13]. The markers to construct the map for population S_1_ had been designed using primers from coding regions in *P*. *trichocarpa* [7]. The QTL was mapped to LG Ib and within the QTL the markers XIII-9_sa, XI_14om_sa and Ro_9_sa showed highest linkage to rust resistance. In *P*. *trichocarpa*, markers XIII-9_sa and XI_14om_sa are located on chromosome 1 while Ro_9_sa is found on chromosome 17 (Fig 1A). Marker Ro_9_sa was designed within a candidate gene for rust resistance in poplar [7].

### BAC library construction and screening

The BAC library was constructed by RxBiosciences Ltd. (Rockville, MD, USA) from DNA extracted from young leaves of the resistant hybrid ‘Björn’ (*S*. *viminalis* × *S*. *schwerinii;* male parent of population S_1_) using the DNeasy Plant kit (Qiagen). DNA was cut with restriction enzyme HindIII and DNA fragments were inserted into cloning vector pIndigoBAC5 with an insert size of 100–150 kb (RxBiosciences Ltd.). The BAC library consisted of 30,720 clones stored in glycerol stocks (in 80 384-well plates), corresponding to 10x genome coverage. The library was divided into two separate parts (each corresponding to 5x genome coverage) and provided with a three level pool system (super-plate pools, plate pools, and row and columns pool), which allowed for screening with PCR-based markers.

Screening of the BAC library was performed for the three map markers closest to the peak of the rust resistance QTL on LG Ib (markers XIII-9_sa, XI_14om_sa, and Ro_9_sa) [13]. Half of the BAC library was used for screening, (i.e. 5x genome coverage). The three markers were located at the peak position of the QTL (within the 1-LOD support interval of the QTL), located within 4 cM according to the linkage map, which correspond to around 500 kb. Considering the size of BAC-clones (100–150 kb each) and possibly alternate starting points in replicated BACs (5x coverage), a large part of genomic region at the peak of the QTL is expected to be covered. Three rounds of PCR reactions on the BAC library pool systems were performed to identify positive BAC clones (following the instructions from RxBiosciences Ltd), where a minimum of 58 PCR reactions was required for each primer pair used (corresponding to a map marker). Once the plate position was identified, the positive clone(s) were cultivated on lysogeny broth (LB) medium (8.7% tryptone, 4.3% yeast extract and 8.7% NaCl in water solution) with chloramphenicol added. DNA was extracted using NucleoBond^®^ BAC 100 purification kit (Macherey-Nagel GmbH & Co. KG, Düren, Germany).

### Solexa sequencing of BAC clones

DNA from all the BAC clones was pooled in equal proportions before sequencing (without labeling individual clones). Sequencing was performed using the Genome Analyzer and Solexa sequencing technology (Illumina Inc., San Diego, CA, USA) at the Dept. of Medical Sciences at Uppsala University. Paired-end reads of 110 bases were produced. The short read sequence data was assembled using *de novo* assembly as implemented in SeqMan NGen software (DNASTAR, Madison, WI, USA). The resulting sequences (contigs) were compared to nucleotide sequence databases using the in-built BLAST (Basic Local Alignment Search Tool) function.

### Gene finding and sanger sequencing

Gene structure of putative resistance genes in the BAC clone assembly was identified using gene prediction software Fgenesh (Softberry, Mount Kisco, NY, USA). PCR primers covering the protein-coding regions of predicted genes were designed with the software Primer3Plus (Dr. Andreas Untergasser, Michelstadt, Germany). PCR was carried out using the following conditions: 10 ng of template DNA was added to a 24 μl mix consisting of H_2_O, 2.5 mM MgCl_2_, 2.5 μl *Taq* buffer (Fermentas, Helsingborg, Sweden) 0.2 mM of each dNTP, 0.25 μM of forward and reverse primers and 1 U of *Taq* polymerase (Fermentas) with: 3 min at 94°C, 35 cycles of (1 min at 94°C, 1 min at 60°C, and 1.5 min at 72°C), and final extension at 72°C for 10 min. The PCR products were separated on 1% agarose gels to confirm fragment size. PCR fragments were sequenced using Sanger method (Macrogen Inc., Seoul, Korea). Single nucleotide polymorphism (SNP) in predicted genes was identified using SeqMan Pro^TM^ version 10.0.1 (3) (DNASTAR) for willow genotypes ‘Björn’ (*S*. *viminalis × S*. *schwerinii*), ‘78183’ (*S*. *viminalis*), ‘Orm’ (*S*. *viminalis*; father of ‘Björn’), ‘79069’ (*S*. *schwerinii*; mother of ‘Björn’).

In order to test for linkage in gene RGA1 between genotypes and phenotypes of the mapping population (S_1_), a SNP marker was designed within the LRR region of the candidate gene (amino acid position 797; synonymous SNP coding for amino acid lycine = K). Ten each of the most susceptible and most resistant individuals in the mapping population were selected based on previous QTL mapping [13], and field rust scores for two years were compared with the SNP marker genotypes.

### Infection experiment for gene expression analysis

Infection experiments for analysis of gene expression were performed on the parents of population S_1_; the resistant variety ‘Björn’ (*S*. *viminalis × S*. *schwerinii*) and the susceptible variety ‘78183’ (*S*. *viminalis*).

In a whole-plant infection experiment, plants of varieties ‘Björn’ and ‘78183’ were cultivated in greenhouse from cuttings for 5 weeks. One day prior to the infection experiment the plants were moved into growth chambers (at 18°C, 16 h day / 8 h night). A high humidity was provided in the chambers by placing the potted plants in wide trays filled with water. Fresh urediniospores of *Melampsora larici-epitea* strain ‘685’ (ITS sequence available in GenBank, accession number JF825969) were used for inoculation. To ensure a high vitality the spores were cultivated shortly before the experiment and mature urediniospores were transferred directly from the leaves on which they were cultivated to the leaves of the experimental plants. A synthetic brush was used to apply the spores to the lower surface of selected and labeled leaves. Fully expanded leaves in the middle part of the plants were used. For each variety, 48 leaves (2 leaves on 24 plants) were inoculated, and 24 leaves (2 leaves on 12 plants) were left non-inoculated as a control. The control plants were kept in a separate growth chamber to prevent the spread of spores from inoculated plants to control plants. Leaf material was sampled from inoculated plants at 4 time-points (6, 12, 24 and 48 hours). Each sample consisted of three pooled leaf discs (11 mm diameter) cut out from a labeled leaf and put into an eppendorf tube containing a 3mm diameter steel ball. The tubes were immediately snap frozen in liquid nitrogen and thereafter stored in a -80°C freezer until RNA extraction. Three biological replicates were collected per condition and time point. Virulence/avirulence pattern of spores was confirmed by visual inspection of remaining infected leaves on the plants 13 days post inoculation.

### Gene expression analysis

The gDNA sequence of gene RGA1 from the BAC clones was used to design qPCR primers and products were designed to span introns allowing us to determine there was no gDNA contamination in our cDNA samples. To normalize the qPCR measurements the reference genes *Actin* and *Ubiquitin* were used, with published primers designed from closely related species [15–16] (S1 Table). These primers were then tested on a number of different cDNA samples from *S*. *viminalis* and the gene products were consistently expressed between samples (data not shown).

Leaf samples from the infection experiment were used in the qPCR analysis. Leaf tissue was disrupted frozen using liquid nitrogen and using 2 mm steel ball in a FastPrep (Biorad, Hercules, CA, USA). Total RNA was isolated from the leaf samples using the Spectrum^TM^ Plant Total RNA Kit (Sigma-Aldrich, St. Louis, MS, USA). First strand-cDNA was synthesized from 1 μg of total RNA, with Maxima First Strand cDNA Synthesis Kit for RT-qPCR (Thermo Scientific, Waltham, MA, USA) according to the manufacturer’s instructions. Real-time PCR was carried out using the first strand cDNA in an iQ5 cycler (Bio-Rad). Maxima Sybr Green/Fluorescein qPCR Master Mix (Fermentas) was used for PCR amplification in a 25 μl total reaction volume consisting of 10 μl of SYBR Green qPCR Master Mix, 0.3 μM forward and reverse primers and 5 ng of cDNA template. All PCRs were performed in triplicate under the following amplification conditions; 10 min at 95°C followed by 40 cycles of 95°C, for 15 s, 60°C for 30 s, and 72°C for 30 s, followed 1 min at 95°C, and melt curve analysis. Expression analysis was performed using Bio-Rad iQ5 –Standard Edition 2.1.97.1001 (Bio-Rad). Significance was calculated using *t*-test and two-way ANOVA.

### Surrounding genomic regions

The DNA sequence from the identified resistance gene (*Rlme1*) in *Salix* population S_1_ was aligned to the genomes of *Populus trichocarpa* v3.0 and *Salix purpurea* v1.0 databases using BLAST (Phytozome 10.2). Predicted genes in the surrounding region 500 kb up- and down-stream of the putative resistance gene were identified using the Phytosome *P*. *trichocarpa* Genome Browser. DNA sequences from genes in *P*. *trichocarpa* annotated as putative resistance genes or containing signature of resistance gene domains were further analysed. Predicted domains were identified using coiled-coil prediction (Andrei Lupas, Princetown University, Princetown, New Jersey, USA), LRRfinder (Victoria Offord, Royal Veterinary College, London, Great Britain) and CD-Search (Aron Marchler-Bauer and Stephen H. Bryant, NCBI, Bethesda, Maryland, USA). Transposons were identified using the software Tandem Repeat Finder (Gary Benson, Boston University, Boston, Massachusetts, USA).

A subset of 10 highly susceptible and 10 highly resistant individuals in *Salix* population S_1_ were selected to test for correlation between resistance phenotypes in population S_1_ and genotypes for new markers designed from R-genes in *P*. *trichocarpa*. Specifically, markers were designed within an R-gene cluster located in the neighborhood of the identified R-gene (RGA1) homolog on chromosome 17 in *P*. *trichocarpa*.

### 3D protein modelling

The predicted LRR region of RGA1 was used to predict tertiary protein structure using Phyre (Lawrence Kelley and Michael Sternberg, Imperial College London, London United Kingdom). The best complete hit was 3OJA, from a complex of LLR containing proteins [17], which was used to build the 3D model of RGA1. Models were then vied in Pymol (The PyMOL Molecular Graphics System, Version 1.7.4 Schrödinger, LLC). Non-synonymous SNPs positions between ‘Björn’ and ‘78183’ were then highlighted and space-filling spheres used to visualise affected amino acids.

## Results

### BAC library screening and gene finding

To identify potential defence-related genes in the QTL region we screened the *S*. *viminalis* × *S*. *schwerinii* BAC library using three markers from the genetic map used in the QTL mapping. The screening resulted in four positive BAC clones, of which three were found for marker Ro_9_sa, and one BAC clone was found mutual for both marker XIII-9_sa and marker XI_14om_sa. *De novo* assembly of the short reads from

Solexa sequencing of the BAC clones resulted in 236 contigs, of which 16 were longer than 2 kb (the longest being 16,858 bases), and the N50 size was 14 kb.

BLAST search of all contigs identified a 3624 bp long DNA sequence on Contig 1 with high similarity to a *P*. *trichocarpa* disease resistance gene (Potri.017G011800) that codes for a resistance protein (PRGDB00062657; formerly XM_002328021). Other BLAST hits in the assembly which related to defense genes were too short or insignificant to be considered for further analysis. When the DNA sequence from Contig 1 was subjected to gene prediction analysis using software Fgenesh, a complete gene containing 7 exons and 6 introns and translated into a protein of 1194 amino acids was identified. Domain finding programs identified TIR, NBS and LRR domains, which together are indicative of a resistance (R) gene. The putative R gene was denoted RGA1 (resistance gene analog 1).

### Polymorphisms in candidate resistance gene

**Fig 2:**
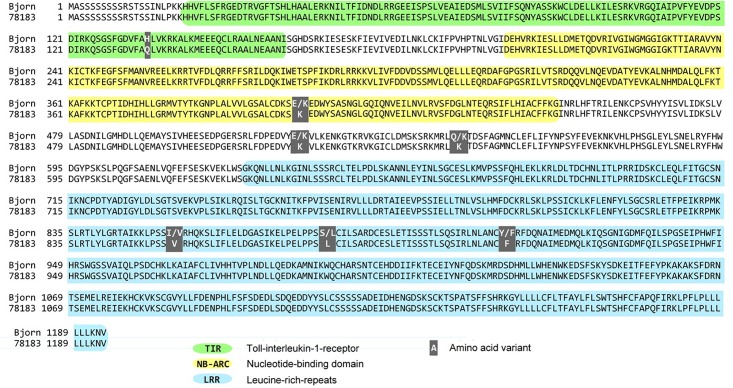
Alignment of RGA1 amino acid sequence from *Salix* genotypes ‘Björn’ (resistant) and ‘78183’ (susceptible). Predicted domains are highlighted. Non-synonymous SNPs are shown with the extent of the amino acid change indicated.

**Fig 3:**
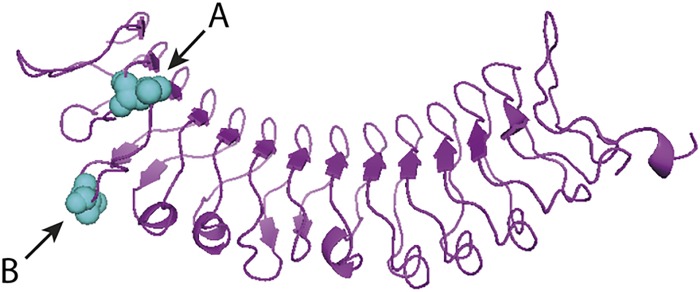
Predicted cartoon tertiary structure of the leucine rich repeat (LRR) of RGA1. Secondary structure shown with flat arrows representing beta sheets, and helices representing alpha helices. A and B are the positions where non-synonymous SNPs between *Salix* varieties ‘Björn’ and ‘78183’ were identified, as indicated by space filling spheres of amino acids (at positions 879 and 853 respectively). The curved structure is common to LRR structures with the internal face involved in protein-protein interactions. The position of SNP A in the internal face of the LRR makes it possible to affect protein binding.

The whole coding region of RGA1 was amplified and sequenced from the susceptible S_1_ parent *S*. *viminalis* ‘78183’ and the resistant hybrid parent *S*. *viminalis* × *S*. *schwerinii* ‘Björn’. The parents of ‘Björn’ (*S*. *schwerinii* ‘79069’ and *S*. *viminalis* ‘Orm’) were also sequenced. Seven non-synomous SNPs were identified between ‘Björn’ and ‘78183’, where ‘Björn’ was heterozygous for six of the seven SNPs. The resulting amino acid sequences are shown in Fig 2, and the corresponding amino acid variants for the parents of ‘Björn’ (*S*. *schwerinii* ‘79069’ and *S*. *viminalis* ‘Orm’) are shown in Table 1. Three SNPs were located in the LRR domain of the RGA1 protein (positions 853, 879 and 910), and in all these three SNPs one of the alleles in hybrid ‘Björn’ showed unique ancestry from *S*. *schwerinii* in this pedigree, i.e. the allelle was present in *S*. *schwerinii* ‘79069’ but not in any of the two pure *S*. *viminalis* (‘Orm’ and ‘78183’). The seven SNPs did alter the properties of the amino acids to various extent, and one of the SNPs in the LRR domain (position 879; changing a serine for a leucine), produced very different properties (e.g. difference in size, hydrophobicity/hydrophilicity, polarity/non-polarity). *De novo* tertiary structural analysis of the LRR domain of the RGA1 protein identified a curved tertiary structure, common to LRR domains (Fig 3) [18]. The location of two of the amino acid changes between ‘Björn’ and ‘78183’ within the LRR are highlighted. The SNP at position 879 (A) changes a serine for a leucine and lies in the inner surface of the curve, while the SNP at position 853 (B) changes an isoleucine for a valine and is on the outer surface.

**Table 1:** Amino acids at non-synonymous SNP sites in gene RGA1 for the parents and two grandparents of mapping population S_1_.

Amino acid position:	135	403	520	547	853	879	910
*S*. *schwerinii* × *S*. *viminalis* ‘Björn’ (S_1_ parent)	H	E/K	E/K	Q/K	I/V	S/L	Y/F
*S*. *viminalis* ‘78183’ (S_1_ parent)	Q	K	K	K	V	L	F
*S*. *schw*erinii ‘79069’ (Björn’s parent)	n.a.	K	K	K	I	S/L	Y
*S*. *viminalis* ‘Orm’ (Björn’s parent)	H	E/K	E/K	Q/K	V	L	F

**Fig 4:**
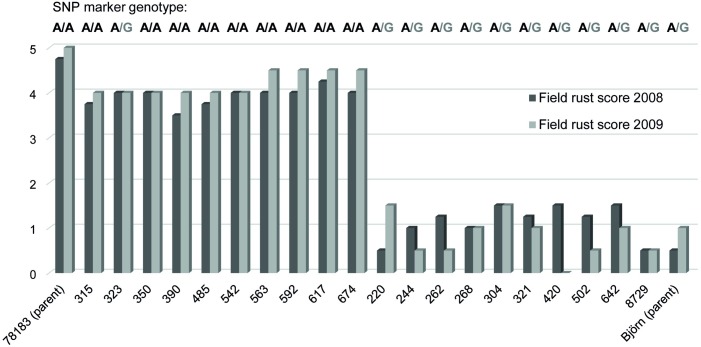
Field rust scores (years 2008 and 2009) and genotype for a SNP marker within gene RGA1 (amino acid position 797) for 10 of the most susceptible and 10 of the most resistant *Salix* individuals in population S_1_. The parents of population S_1_ (*S*. *viminalis* ‘78183’ and *S*. *viminalis* × *S*. *schwerinii* ‘Björn’) are also included.

Linkage between rust resistance phenotypes and genotypes of a synonymous SNP marker (position 797) were tested for ten of the most susceptible and ten of the most resistant *Salix* individuals in mapping population S_1_ (Fig 4). All the resistant individuals had genotype A/G, while all but one (variety 323) of the susceptible individulas had genotype A/A. The susceptibe *S*. *viminalis* parent ‘78183’ also had genotype A/A, while the resistant hybrid parent ‘Björn’ had genotype A/G (where G is inherited from the *S*. *schwerinii* grandparent ‘79069’).

### Gene expression

**Fig 5:**
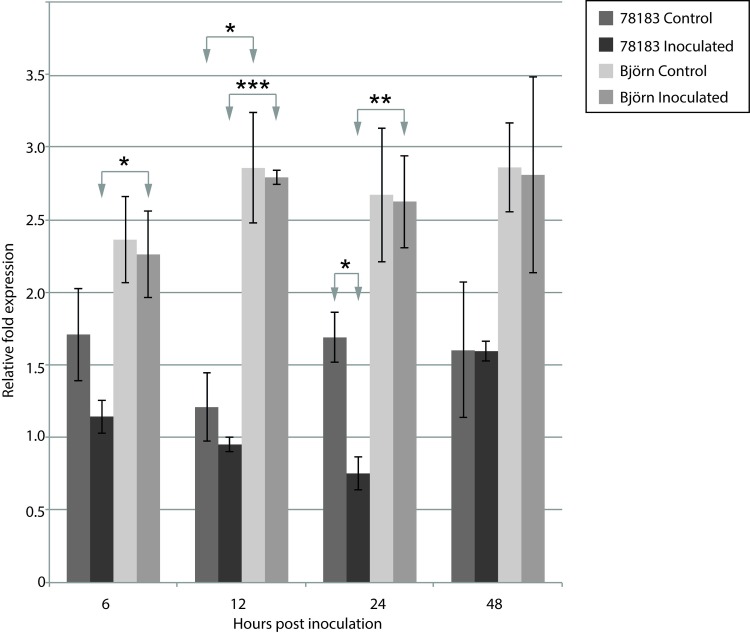
Gene expression data for gene RGA1 in susceptible (‘78183’) and resistant (‘Björn’) *Salix* genotypes when challenged with rust spores, at 6, 12, 24 and 48 hours post inoculation. Relative fold expression is shown for control and inoculated plants with three biological replicates for each sample and condition. * indicates significance at p = 0.05. Error bars indicate standard deviation. P-values for significant differences between genotypes and between treatments are indicated: * p<0.05; ** p<0.01; *** p<0.001.

**Fig 6:**
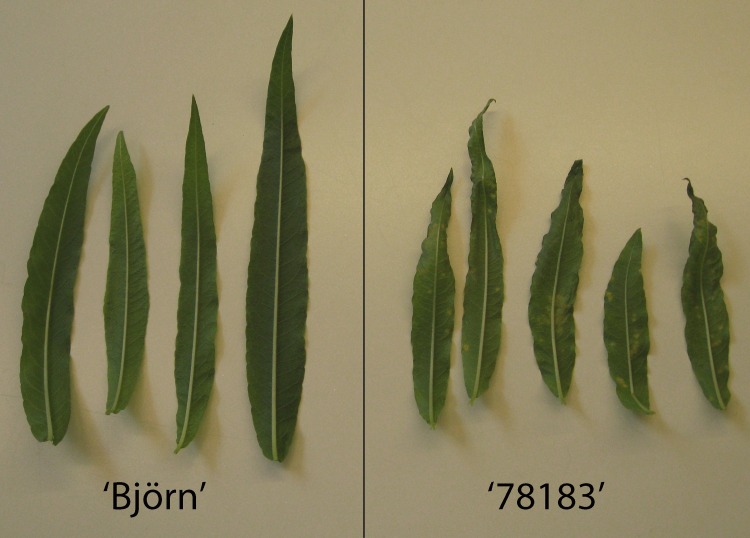
Infection pattern on inoculated leaves from the gene expression experiment 13 days post inoculation. Leaves from the resistant *Salix* genotype ‘Björn’ to the left, and the susceptible genotype ‘78183’ to the right.

Gene expression levels of RGA1 in both resistant and susceptible genotypes were tested using quantitative real time PCR (qPCR) on whole attached leaves. Overall gene expression was significantly higher in the resistant genotype ‘Björn’ compared to the susceptible genotype ‘78183’ in control (p<0.005) as well as inoculated plants (p<0.005) (Fig 4) using pooled time points to increase sample size. When the time points were analyzed separately, the genotypes had significantly different expression in inoculated plants at 6, 12 and 24 hours post inoculation (hpi), while for control plants there was only a significant difference at 12 hpi (Fig 5). There was no significant difference in the resistant genotype ‘Björn’ between control and inoculated plants at any time point (Fig 5). In the susceptible genotype ‘78183’ there was a lower expression in the inoculated compared to control plants at time points 6, 12, and 24 hpi, however only at 24 hpi was the difference statistically significant (p<0.02). At 48 hpi expression levels were similar between inoculated and control plants. Fig 6 shows remaining leaves from inoculated plants 13 days post inoculation. Several uredinia were present on all inoculated leaves on the susceptible variety ‘78183’, while there was no rust on the leaves on the resistant variety ‘Björn’.

### Surrounding genomic regions

By BLAST searches in the *P*. *trichocarpa* (*Ptr*) and *S*. *purpurea* (*Sapur*) genomes we found a *Ptr* homolog of RGA1 on chromosome 17 (gene Potri.017G011800; max-score = 6674; e-value = 0.0), and a *Sapur* homolog on Scaffold0941 (SapurV1A.0941s0040; max-score = 6876; e-value = 0.0) (Fig 1B and 1C, gene *D*). In tandem with the RGA1 homologs, in opposite orientation, we found a gene coding a protein with Jacalin-like lectin domains (Potri.017G011900) (gene *E*), both in *Ptr* chromosome 17 and in *Sapur* Scaffold0941 (SapurV1A.0941s0050), as well as in Contig 1 from the BAC clone sequences (i.e. LG Ib in *Salix* population S_1_). Using the Tandem Repeat Finder software, a putative retrotransposon was found between the two genes in *Sapur* Scaffold0941.

Reciprocal BLAST searches with the *Ptr* RGA1 homolog (Potri.017G011800; gene *D*) and adjacent genes (*A*, *B*, *C*, *F*, *G*, *H*, *I* and *J*) (Fig 1B and 1C) against the *Ptr* genome have best hits for Potri.017G010800 (gene *D′*) and genes *A′*, *B′*, *C′*, *E′*, *F′*, *G′*, *H′*, *I′* and *J′* in the same orientation and position directly upstream of the *Ptr* RGA1 homolog. This indicates that in *P*. *trichocarpa* the 80 kb region containing genes *A-J* on chromosome 17 has undergone a recent duplication. Using genes *A-J* from the *Ptr* genome to blast the *S*. *pupurea* genome, the genes *A*, *B*, *C*, *F*, *G*, *H*, *I* and *J* gave best blast hits on *S*. *pupurea* chromosome 17, however the region corresponding to gene homologs *D* and *E* (the *Sapur* RGA1 homolog and the Jacalin domain-containing gene, respectively) was absent on *Sapur* chromosome 17. The 5 other genes (*K-N*) present on *Sapur* Scaffold0941 were used to blast the *Ptr* genome, where the best hits were found on the end of chromosome 1 from 48,150 kbp to 48,259 kbp (Fig 1C).

The *Ptr* genome was used to search for genes related to disease resistance further up- and downstream of the *Ptr* RGA1 homolog. No resistance-related genes were identified in the region upstream of the RGA1 homolog, while a cluster of 9 putative R-genes containing NBS-LRR domains were identified in a region approximately 200 kb downstream on chromosome 17. In order to investigate if a similar R-gene cluster could be present in the QTL region on LG Ib in *Salix* population S_1_, we tested if polymorphic markers designed within the gene cluster were linked to resistant/susceptible phenotypes in a subset of genotypes in population S_1_. No correlation was found, indicating that any similar R-gene cluster was not linked to rust resistance and thus probably not present on LG Ib in *Salix* population S_1_ (data not shown).

## Discussion

Using a genomic BAC library we identified a complete putative resistance gene, RGA1, of type TIR-NBS-LRR close to a major QTL for rust resistance in a *S*. *viminalis* × (*S*. *viminalis* × *S*. *schwerinii*) family. Several resistance components were associated to this major QTL, among them necrotic flecking which is an indication of hypersensitivity response. Such a reaction, together with large effects on all other resistance components, is likely to be governed by a NBS-LRR type of gene that is involved in pathogen recognition and acting at an early stage of infection. Therefore the structure of RGA1 makes it a plausible candidate resistance gene for this major QTL. It should however be acknowledged that the full-length genomic sequence in the QTL region may not have been captured in the BAC library screening, meaning that there is a possibility that any additional resistance genes could have remained undetected.

The previously identified major QTL accounted for up to 56% of the variation in resistance traits [13] suggesting that the responsible gene contains an important allele or mutation. Seven non-synonymous SNPs in the RGA1 gene were identified between the susceptible and resistant parental genotypes. Of these, five changed the amino acid properties to an extent that may affect the protein function. Three were located in the LRR domain where one changed a hydrophilic serine to a hydrophobic leucine. In each of these three SNPs, one amino acid variant (allele) showed unique ancestry from *S*. *schwerinii* in the hybrid ‘Björn’. The LRR is a receptor domain and in R-genes known to interact with proteins or molecules produced when a plant is under attack from a pathogen or bind cofactors that are released when a pathogen is detected [12, 19]. This initiates a defence response and produces a resistant phenotype. The LRR domain of R-genes can vary but maintains a repeat pattern of leucine residues and this variation can aid recognition of rapidly evolving pathogens [20]. LRRs form a curved structure that is involved in interactions with either pathogen components directly or indirectly though protein complexes [21]. Ellis et al. [22] showed that polymorphism in the LRR region of a NBS-LRR resistance gene in flax is necessary for specificity towards avirulence genes in *Melampsora lini*. Binding of the avirulence proteins is predicted to occur within the concave part of the LRR [23]. In gene RGA1, the three SNPs within the LRR between the resistant and susceptible genotypes may alter the binding domain, thus changing the interaction with potential targets. Computer modelling suggests that one SNP in gene RGA1 is located on the concave surface of the LRR and therefore supports the hypothesis that it may alter protein interactions. It should be acknowledged that also SNPs occurring in other domains could be responsible for the observed differences in rust resistance phenotypes. For example, SNPs might affect nucleotide binding in the NB-ARC domain and thus regulation of R-protein activity [19].

A high degree of linkage between genotypes of a SNP marker located within the LRR domain of RGA1 (amino acid position 797) and rust resistance phenotypes supports the plausibility for RGA1 as a candidate resistance gene.

Gene expression of RGA1 was significantly different between the susceptible and resistant genotypes, even in control conditions where plants were not inoculated with *M*. *larici-epitea*. It has been shown in other model systems that R-gene expression level can be responsible for susceptible/resistant phenotypes [24–25]. If expression is below a critical level the pathogen may be able to avoid detection and successfully cause infection. The reduction in expression when challenged with the pathogen after 24 hours in the susceptible genotype suggests that there is an interaction between the pathogen and RGA1 expression levels in inoculated cells. In a successful defence response the plant will recognise the presence of the pathogen and initiate defence responses mechanisms such as thickening of the cell wall, the hypersensitive response and systemic acquired resistance. If the recognition is unsuccessful and there is a delay in the initiation of defence responses it can lead to infection. The reduction in expression levels of RGA1 in inoculated susceptible tissue suggests the pathogen is affecting the mRNA levels through the secretion of effectors, giving rise to effector-triggered susceptibility (ETS) [12]. In the closely related *P*. *trichocarpa*–*M*. *larici-populina* pathosystem, the fungus has penetrated the leaf and spread in the leaf tissue at 24 hpi [26], and it is about the time of formation of the first haustoria [27]. Besides being the fungus’ nutrient acquisition structure inside the cells, haustoria are also the main source of effector delivery [28]. In the resistant genotype there is no up-regulation of RGA1 after inoculation, which indicates that the gene is not involved in an active response initiated after the pathogen has been recognized, such as seen in many pathogenesis-related genes, for example in *Populus* after inoculation with *Melampsora* rust fungi [26, 29]. Our results showed that RGA1 is constitutively expressed in both susceptible and resistant genotypes and suggest that a critical level of expression may be required for the plant to produce a resistant phenotype.

The identification of a differentially expressed gene with a classic R-gene domain structure within the QTL region relating to rust resistance is exciting. The fact that the gene is expressed at a lower level in the susceptible genotype compared to the resistant suggests that the difference might be in the non-coding regulatory region upstream of the gene. The down-regulation at 24 hpi in the susceptible genotype suggests that the pathogen might be actively interrupting the expression levels. Looking at the expression profiles of other R-genes in the genome would make it possible to determine if this is unique to this gene, or if down regulation is a feature within a number of genes in the susceptible genotype. As a transformation protocol is unavailable for *S*. *viminalis* and therefore functional analysis is not possible, it is currently hard to determine if this gene alone, or any of the SNPs identified, are responsible for the resistance phenotype. Functional screening of *Salix* more axillary growth (MAX) genes in *Arabidopsis* has been previously performed [30] but due to the high host specificity for the rust pathogen it is unlikely that RGA1 could be screened in this way. When a transformation method becomes available for *S*. *viminalis* it will be extremely interesting to study the RGA1 and other potential R-genes to understand further the interactions in the *Melampsora*-*Salix* pathosystem.

The QTL associated with rust resistance has been identified towards the end of LG Ib by Samils et al. [13] corresponding to the orthologous *P*. *trichocarpa* chromosome 1. Most of the markers in or around the QTL region agree with this, however marker Ro_9_sa, which is located on LG Ib in *Salix* population S_1_ and tightly linked to resistance, is located on Chromosome 17 in *P*. *trichocarpa*. In *Salix* population S_1,_ this marker is located in the putative R-gene, which we termed RGA1, a close ortholog of *P*. *trichocarpa* Potri.017G011800. This indicated that since the split of *Populus* and *Salix* the gene may have jumped from chromosome 1 to 17 or vice versa in one of the genera. This is supported by the fact that *Salix* homologs of a cluster of resistance genes on chromosome 17 downstream of the RGA1 homolog in poplar were not linked to the resistant phenotype in *Salix*.

The large cluster of R-genes found upstream on *Populus* chromosome 1 show high level of homology (e-value = 0.0) with one another, which suggests recent large-scale duplication events. No hits were found for this cluster in the BAC clones from the QTL region. The best blast hits in *S*. *purpurea* were numerous and scattered over chromosome 16 and 8 scaffolds. The draft nature of the *S*. *purpurea* genome allows a limited analysis of the genomic region within the QTL, however using the *Populus* genome as a reference gives us insights to potential R-genes. Due to the fast evolving nature of R-genes it is likely that even if chromosome structure are similar between *Salix* and *Populus*, there may be large variation between the R-genes location and number. Poplar has been shown to have a high number of R-genes, over twice that of *Arabidopsis thaliana* [8, 9]. This could be due to recent duplications such as that seen in the 80 kb tandem duplication in *P*. *trichocarpa* containing Potri.017G011800 and nine surrounding genes on chromosome 17. The number of resistance genes in *Salix* is currently unreported but with the unpublished *S*. *purpurea* genome available, a genome wide analysis will surely be presented soon. This will allow detailed comparisons between *Salix* and *Populus* R-genes but also with other species and further our understanding of rust resistance in *Salix*

In this study we identified a candidate resistance gene, RGA1, located in a major rust resistance QTL with inheritance from the Asian species *S*. *schwerinii*. RGA1 showed a lower constitutive expression in a susceptible *Salix* genotype than a resistant, and there were also indications that the rust pathogen *M*. *larici-epitea* may actively suppress RGA1 gene expression in the compatible interaction. It remains to further validate the candidate resistance gene and to develop DNA markers for tracking the gene in various genetic backgrounds in marker-assisted breeding.

## Supporting Information

S1 Table Primers used in qPCR analysis. (DOCX)
