# Supplementary material for: Identification of a Differentially Expressed TIR-NBS-LRR Gene in a Major QTL Associated to Leaf Rust Resistance in Salix
Source: PLoS One. 2016 Dec 21;11(12):e0168776. doi: 10.1371/journal.pone.0168776 (PMC5176316; doi:10.1371/journal.pone.0168776)
Supplement: S1 Table — (DOCX) [file pone.0168776.s001.docx]

S1 Table. Primers used in qPCR analysis.

| Gene | Forward sequence | Reverse sequence | Product size |
| --- | --- | --- | --- |
| *Rmle1* | TGAACGTAGCAGGCTGTTTG | CCTCATTTTCCTTGATTTGGA | 159 |
| *Actin* | AGCGTATGTTGCCCTTGACTACGA | TTCCGATGAGAGATGGCTGGAAGA | 156 |
| *Ubiquitin* | CCAAGCCCAAGAAGATCAAGC | GCACCGCACTCAGCATTAGG | 127 |
